# Comparing different revisions of the Childhood Health Assessment Questionnaire to reduce the ceiling effect and improve score distribution: Data from a multi-center European cohort study of children with JIA

**DOI:** 10.1186/1546-0096-8-16

**Published:** 2010-05-17

**Authors:** W Groen, E Ünal, M Nørgaard, S Maillard, J Scott, K Berggren, E Sandstedt, M Stavrakidou, J van der Net

**Affiliations:** 1 Child Development and Exercise Centre, University Hospital for Children and Youth 'Het Wilhelmina Kinderziekenhuis' University Medical Centre Utrecht, Utrecht, the Netherlands; 2 Physical Therapy and Rehabilitation Department, Hacettepe University, Ankara, Turkey; 3 Department of Physiotherapy, Århus University Hospital, Skejby, Århus, Denmark; 4 Institute of Child Health, Great Ormond Street Childrens Hospital, London, UK; 5 Birmingham Children's Hospital NHS Foundation Trust, Birmingham, UK; 6 Astrid Lindgren Children's Hospital, Karolinska University Hospital, Stockholm, Sweden; 7 Department of Physiotherapy, The Queen Silvia Children's Hospital, Göteborg, Sweden; 8 Pediatric Immunology and Rheumatology Referral Center First Dept of Pediatrics, Ippokration General Hospital, Thessaloniki, Greece

## Abstract

**Background:**

The original version of the Childhood Health Assessment Questionnaire (CHAQ30orig) suffers from a ceiling effect and hence has reduced clinical validity. The purpose of this study was to evaluate the effect of adding eight more demanding items (CHAQ38) and a new categorical response option (CATII) on discriminant validity and score distribution in a European patient sample.

**Methods:**

Eighty-nine children with Juvenile Idiopathic arthritis (JIA) and 22 healthy controls, aged 7-16 years, were recruited from eight centres across Europe. Eight new CHAQ items and scoring option were translated back and forth for the countries in which they were not already present. Demographic, clinical, and CHAQ data were collected on-site. Subsequently, five different scoring methods were applied, i.e. the original method (CHAQ30orig) and four alternatives. These alternatives consisted of the mean item scores for the 30 and 38-question versions with either the original (CATI), or the new categorical response option (CATII). The five versions were tested for their ability to distinguish between patients and controls. Furthermore score distributions were evaluated and visualized by box and whisker plots.

**Results:**

Two CHAQ revisions with the new response option showed poor discriminative ability, whereas one revised version (CHAQ38CATI) had comparable discriminative ability comparable to the original CHAQ. A profound ceiling effect was observed in the original scoring method of the CHAQ (27%). The addition of eight more demanding items and application of a plain mean item score reduced this significantly to 14% (χ^2 ^= 4.21; p < 0.05).

**Conclusions:**

Revising the CHAQ by adding eight more demanding items and applying a plain mean item scoring (CHAQ38CATI) maintained discriminant ability and reduced the ceiling effect in a European patient sample. The new categorical response option (CATII) seemed promising, but was less able to distinguish children with JIA from healthy controls and had less favourable distribution characteristics. The CHAQ38CATI is advocated for future use in mildly affected JIA patients.

## Background

The Childhood Health Assessment Questionnaire (CHAQ) is the most widely used functional health status measure in children with Juvenile Idiopathic Arthritis (JIA)[1,2]. It assesses functional ability in 8 domains of physical function (30 items) for children between the ages of 6 months up to 18 years. Each item is scored on a four point scale ranging from 0 (without any difficulty), 1 (with some difficulty), 2 (with much difficulty), 3 (unable to do). Utilization of assistance and or aids in a domain sets the score to a minimum of 2 for that domain. The mean score of the eight domains finally makes up the disability index and ranges from 0 (no disability) to 3 (disabled)[3]. The disability index is supplemented with two visual analogue scale (VAS) scores: one for pain, and one for global assessment of overall well being.

Although originally designed to be used as an outcome measure in childhood rheumatic diseases, the CHAQ is currently used in other conditions such as spina bifida and juvenile dermatomyositis[4,5]. Over the past decade, as a result of improved treatment strategies [6], the CHAQ suffers from a ceiling effect. The most noticeable consequence of this ceiling effect is that it is impossible to measure improvements at the better end of the functional spectrum. In other words, clinical validity is reduced.

For this reason researchers have recently took up the challenge to introduce and test suggestions for revision of this instrument to improve its psychometric properties. Lam et al. [7], for example, examined the discriminative validity and score distribution of three new versions of the CHAQ. One important aspect of these versions was ignoring both the domain structure and use of aids and assistance. These new versions proved to enhance discriminative validity of the CHAQ in a Canadian cohort of children with a wide diversity of musculoskeletal conditions. In addition, Takken et al. [8] performed thorough statistical procedures and concluded that two manoeuvres could enhance the psychometric properties of the CHAQ. Firstly, by removing twelve redundant items, and secondly, by ignoring the domain structure and the use of aids and assistance. In addition, a recent cohort study in 2663 patients showed that removal of aids and assistance from the CHAQ did not change the interpretation of disability at a group level. Therefore the authors conclude that a CHAQ without these items is a more feasible and valid alternative for the evaluation of disability in JIA patients [9].

The encouraging findings of Lam et al. have resulted in efforts to replicate them in Dutch populations. Ouwerkerk et al. [10] examined the score distribution of several revised versions of the CHAQ in a partial retrospective study. In extension of this work Van Dijk et al. [11] examined the score distributions of new versions prospectively in children with JIA, in a Dutch multi centre study. Results of these studies are comparable to those of Lam et al. and encouraged further investigation into a broader cross cultural context. As a result, the aim of this prospective cross-sectional European multi centre study was to explore the score distribution of 4 different versions of the CHAQ in different European languages. Three revisions of the CHAQ were of major interest in the current study, namely, 1) the addition of 8 more challenging items, 2) ignoring the scale structure and aids and assistance, and 3) the effect of a new categorical response option, that allows patients to rate themselves not only worse than their peers, but even better at activities than most other children and young people their age.

Our specific hypothesis is that the addition of the 8 more challenging items and a new response option enhances discriminative validity and has a positive influence on the score distribution of the CHAQ compared to the original CHAQ.

## Methods

### Patients

This study was a collaboration of eight centres across Europe, i.e., the Netherlands (Utrecht), United Kingdom (London and Birmingham), Greece (Thessaloniki), Turkey (Ankara), Denmark (Århus) and Sweden (Stockholm and Göteborg). All collaborators were participants of the Pediatric Rheumatology European Society (PReS) health professional research effort. They were invited to provide data of a convenience sample of 10-15 children, aged 7 to 16, who are diagnosed with JIA according to the ILAR criteria[12].

### Questionnaires and procedure

**Figure 1:**
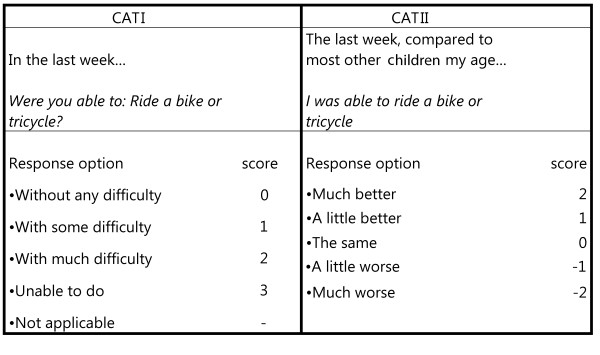
**Examples of the two categorical response options**.

First, the 8 more demanding items (as shown in Appendix) and the new categorical response option, on which children compare their ability to most other children their age (Figure 1; right column), were translated forward into Swedish, Turkish, Danish and Greek language. The translations were performed on-site by academic-level personnel. Each participating centre chose two of the most appropriate translators: one for forward and one for backward translation. The back translations from these four centres (English versions) were then compared with the original of Lam and colleagues[7]. This ultimately resulted in Greek, Turkish, Swedish and Danish translations (Additional file 1). A Dutch translation had already been adapted and there was no need for another UK- English translation[10].

Then, a worksheet was constructed including the original CHAQ of Singh et al. [3] extended with the 8 more challenging items (Appendix). For every item a score was obtained by two scoring methods. Firstly by the original scoring method ranging from without any difficulty (0) to unable to do (3) (Figure 1; left column). Secondly by the new scoring method ranging from much worse (-2) to much better compared to most other children your age (2) (Figure 1; right column). In this way all possible CHAQ revisions were together in one comprehensive document. This facilitated the completion of the forms by physiotherapist or occupational therapist at the different sites. The first page of this worksheet contained questions about demographic (gender, age) and clinical data (e.g. medication and joint involvement). Active disease and remission were defined as follows: active disease was defined as the presence of elevated erythrocyte sedimentation rate and swollen joints that are painful or have a limited range of motion. Remission, on the other hand, was defined by the absence of these features for 6 months or more (either with or without medication). Joint involvement was defined as a joint that was painful and/or swollen (as measured by palpation) and/or limited in range of motion (as measured by goniometry). Joint involvement was evaluated by pediatric physical therapists on site.

**Figure 2:**
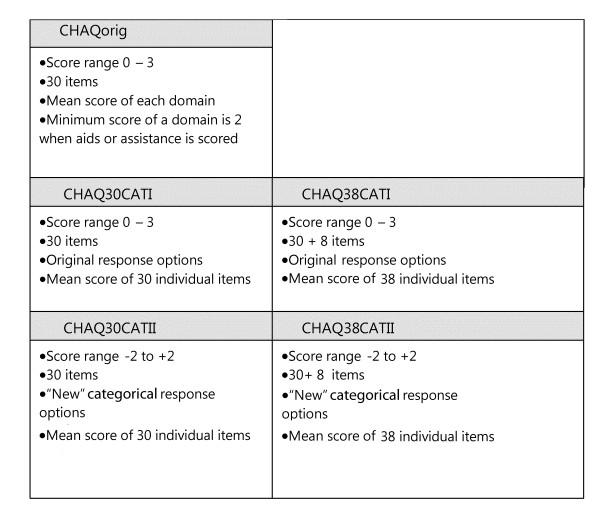
**Score details and calculation methods of the original and revised CHAQ scores**.

Afterwards, five different scores were obtained from the worksheets: the original score by Singh et al. (CHAQ30orig), and the mean item scores for the 30 and 38-question versions with two categorical response options each. This resulted in the Chaq30CATI and II and Chaq38 CATI and II scoring methods. Figure 2 summarizes details about scoring range, response options and method of calculating a total score.

The study was approved by the board of ethics of the University Medical Center Utrecht and was consequently adopted by all local boards of all collaborating centers.

### Statistical analyses

All analyses were performed using SPSS version 15. Score distribution of the CHAQ versions were presented by descriptive statistics (median scores, percentile scores, interquartile range (IQR) and % of scores at ceiling). A ceiling effect was defined by 15% or more patients scoring the best possible score[13]. Box-and-whisker plots were used to visualize score distributions. Score distribution of the CHAQ versions were tested for normality by the Kolmogorov-Smirnov (K-S) one-sample test of normality. Differences in CHAQ scores between revisions and the original were compared by Wilcoxon ranked sign test. Differences in CHAQ scores between JIA patients and controls were compared for each version by a T-test for unpaired samples as well as by Mann-whitney U test. Discriminative validity of the CHAQ revisions was determined by using the relative efficiency statistic as defined by the ratio of squared t-scores. A higher ratio represents a higher discriminative validity[7]. A p-value of < 0.05 was considered significant.

## Results

Data of 89 patients as well as 22 healthy controls were available for analysis. Demographic and clinical data of all subjects are presented in Table 1. There were some missing data on the worksheets (disease activity (n = 2) and diagnosis (n = 7)), and repetitive attempts to retrieve the information from the corresponding study sites have failed. Characteristics of score distributions of the CHAQ and four revisions are presented in Table 2. All distributions differed significantly from a normal distribution. Some minor differences in KS-statistics were noticed in favour of the 38 items versions (lower scores reflect a more normal distribution of the scores). The positive scores for skewness of all scoring methods indicate a shift of the peak to the left of the expected normal distribution within the scoring range, indicating that the children only suffered from mild disability. Positive scores for kurtosis as observed in the CATI versions indicate that, when compared with a normal distribution, relatively more scores are in the peak, than in the tails of the distribution. On the other hand, a negative score indicates that relatively more scores are in the tails, than in the peak.

**Table 1:** Demographic and clinical data and scores of the five CHAQ versions of patients and healthy controls.

	JIA	Healthy	p-value
n	89	22	
Age (years)	11.9 (2.7)	12.0 (2.7)	0.67
Sex (m/f)	19/70	12/10	
Diagnosis (n =)			
PA	40		
SA	10		
PO	17		
EO	9		
OL	6		
Missing	7		
Disease duration (years)	5.4 (3.6)		
Disease activity (n = (%))			
Active	38 (43)		
Remission	49 (55)		
Missing	2 (2)		
Medication (n = (%))			
None	14 (16)		
NSAID	43 (48)		
MTX	46 (52)		
Prednisolone	9 (10)		
Biologicals	24 (27)		
Joint Involvement (median (IQR))	10 (10-30)		
VAS Pain	2.7 (0-9.5)		
VAS GA	2.5 (0-10)		
CHAQ30orig	0.38 (0-2.13)		

Scores for VAS and CHAQ are presented as median and range. Values for age and disease duration are presented as mean and standard deviation.

Polyarthritis; SA, Systemic Arthritis; PO, Persistent Oligoarthritis; EO, Extended Oligoarthritis; OL, Oligoartrhitis; VAS GA: Visual analogue scale global assessment of overall wellbeing. Joint involvement: percentage of joints affected by JIA.

**Table 2:** Score distribution characteristics of the five CHAQ versions.

CHAQ Version	Median (range)	% at ceiling	KS-statistic	*p*	IQR	Skewness	Kurtosis
CHAQ30orig	0.38 (0-2.13)	27*	0.19	<0.001	1.06	0.85	-0.51
CHAQ30CATI	0.10 (0-1.62)^a, c^	27*	0.22	<0.001	0.40	1.62	2.65
CHAQ38CATI	0.19 (0-1.84)^a, b^	14	0.19	<0.001	0.49	1.42	1.69
CHAQ30CATII	-0.033 (-1.69-2)^e^	1	0.24	<0.001	0.35	0.94	5.43
CHAQ38CATII	-0.12 (-1.7-1.76)^d^	0	0.20	<0.001	0.45	0.72	3.60

* ceiling effect, > 15% at ceiling; IQR: Interquartile range, KS: Kolmogorov Smirnov; *p*: probability value of the KS one sample test of normality.

^a ^significantly different from CHAQ30orig, Wilcoxon p < 0.001

^b ^significantly different from CHAQ30CATI, Wilcoxon p < 0.001

^c ^significantly different from CHAQ38CATI, Wilcoxon p < 0.001

^d ^significantly different from CHAQ30CATII, Wilcoxon p < 0.001

^e ^significantly different from CHAQ38CATII, Wilcoxon p < 0.001

Three revised versions were less able to distinguish between patients and controls than the original version (lower values for relative efficiency, Table 3). The CATII versions showed clearly lower values for relative efficiency than the revised versions with the original response option (CATI versions). The CHAQ38CATI was nearly as efficient as the original version (relative efficiency of 1.01).

**Table 3:** Discriminative validity of different CHAQ revisions compared to the original CHAQ.

			T-test		Mann-Whitney U-test			
					
CHAQ version	Score patients	Score controls	T statistic	P	U statistic	Z score	P	relative efficiency
CHAQ30orig	0.38 (0-2.13)	0 (0-0.63)	-7.52 (df 109)	<0.001	344	-4.84	<0.001	1.00
CHAQ30CATI	0.10 (0-1.62)	0 (0-0.23)	-6.90 (df 102)	<0.001	333	-4.92	<0.001	0.84
CHAQ38CATI	0.19 (0-1.84)	0 (0-0.26)	-7.56 (df 101)	<0.001	263	-5.33	<0.001	1.01
CHAQ30CATII	-0.033 (-1.69-2)	0 (-0.026-0.071)	2.37 (df 90)	0.02	509	-3.54	<0.001	0.10
CHAQ38CATII	-0.12 (-1.7-1.76)	0.013 (-0.052-0.42)	3.78 (df 107)	<0.001	403	-4.28	<0.001	0.25

CHAQ scores for patients and controls are shown as median and range. Relative efficiency is the ratio of squared t-statistic with the original CHAQ (chaq30orig) as reference.

The addition of 8 challenging items reduced the number of children achieving the highest possible score for the CATI version (from 27 to 14%; χ^2 ^= 4.21; p < 0.05). It also increased the relative efficiency to distinguish patients from controls for both CATI (from 0.84 to 1.01) and CATII versions (from 0.10 to 0.25; Table 3).

**Figure 3:**
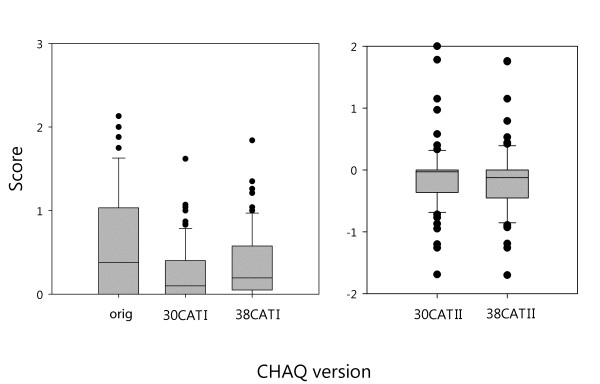
**Box and whisker plots of the scores on the original and revised versions of the CHAQ**. The box contains 50% of all values (the 25^th ^to 75^th ^percentile) and is divided by the horizontal bar which is the median value (50^th ^percentile). The whiskers show the remainder of the distribution (1.5 × Inter Quartile Range). Outliers are shown as dots.

The box plots in Figure 3 illustrate the score distribution of the 5 versions of the CHAQ. Both 30CATI and 38CATI versions show lower scores than the original scoring options. In addition, the 38CATI version shows significant higher scores than the 30CATI version (higher scores on CATI means worse functional ability). On the other hand, scores of the 38CATII version were significantly lower than those of the 30CATII (lower scores on CATII means worse functional ability) (p < 0.01 for all).

## Discussion

This study was the first to examine the scores of revised versions of the original CHAQ across Europe. The aim was to examine the effect of the addition of 8 more challenging questions and a new categorical response option on discriminant validity, score distribution and ceiling effect of the CHAQ.

In accordance with our hypothesis, the addition of 8 more challenging questions reduces the number of patients at the ceiling in the CatI scoring option (from 27 to 14%). This finding is in accordance with the study of Ouwerkerk et al. [10], Lam et al. [7] and Van Dijk et al. [11] that both show a reduced number of patients at the ceiling.

The addition of 8 more challenging questions also results in more normally distributed data compared to the scoring options with 30 questions (KS statistic of 0.19 and 0.20 v.s. 0.22 and 0.24). These findings are in accordance with the study of Lam et al. [7]. Moreover it broadened the score range as is reflected by higher IQR values (0.49 and 0.45 v.s. 0.40 and 0.35), which leads to better discrimination between patients and controls. The original scoring method had a larger IQR and a higher discriminative ability than the CATI versions. On the other hand it showed more patients scoring the best possible score, making this score option a less desirable one. Moreover, the CHAQ38CATI was almost as efficient in distinguishing JIA and healthy as the original version. Moreover, the CHAQ38CATI does not suffer from two negative effects, namely the lack sensitivity to improvement over time and the punishing effect of aids and assistance that is inherent to the domain score structure of the original CHAQ [8].

It was surprising to see that the revised versions did not discriminate patients and controls to the same extent as in the study of Lam et al. [7] This could be a result of the relative homogenous patient sample in our study. In the study of Lam et al. a wider spectrum of musculoskeletal conditions was studied. One implication of this finding could be that for JIA patients that are at the mild end of the disability spectrum, the CATII revisions are not recommended. The discriminative ability of the CATII versions was even less than the original CHAQ, which is undesirable because more patients have to be included in clinical trials.

The application of a new categorical response option (CATII, see Figure 1) yielded data that was less normally distributed then the CATI response option as reflected by lower KS values. Furthermore the score distributions for the CATII options were narrower than the original and CATI options as was reflected by higher kurtosis and lower IQR values. One remarkable finding was that one patient rated his/herself as much better than other children their age for all items. We don't know the exact cause of this, but it shows a potential weakness of this scoring method.

One possible limitation of the current study is that we used interviews to gather the CHAQ data, whereas current knowledge of the psychometric properties of CHAQ is based on parent and child report. We are aware that this could have introduced some bias, however, a study of Verrips et al. [14] showed moderate agreement between quality of life questionnaires completed by mail compared with face to face interviews. Even higher agreement was reported for the motor domain of the HRQOL with ICC's ranging from of 0.7 - 0.81. Therefore we are confident that the bias of interviewing the patients is minor. Another limitation is that the group of JIA patients in this study was relatively homogeneous in terms of level of disability. This could limit generalizability of the results to patients with higher levels of disability. As the CHAQ is a core outcome variable in Europe it is important to confirm these preliminary findings in future studies with larger cohorts.

## Conclusions

In summary, in this study we examined the effect of four revised versions of the CHAQ on score distribution. Revising the CHAQ by adding eight more demanding items and applying a plain mean item scoring reduced the ceiling effect. The application of a new categorical response option, although theoretically appealing, showed some disappointing results: a low discrimative ability, a narrow score distribution and a considerable deviation from a normal distribution. Of the five versions studied, the CHAQ38CATI seems most favourable for use in mildly affected JIA patients.

## Competing interests

The authors declare that they have no competing interests.

## Authors' contributions

WG and JN designed the study. EU, KB, ES, MN, and MS performed the translation procedures of the revised CHAQ versions. EU, JN, SM, JS, KB, MN, ES, and MS performed on-site patient recruitment. WG performed statistical analyses and drafted the manuscript. All authors read and approved the final manuscript.

## Appendix

Eight more challenging items of the CHAQ38

31. I think I could have done climbing activities by myself (examples: climbing trees, rocks, or climbing over a fence).

32. I think I could have played team sports with others in my class (examples: basketball, baseball, soccer, hockey).

33. I think I could have played some sports by myself or with a few friends (examples: dribbling and shooting basketball).

34. I think I could have played team sports in competitive leagues (examples: local basketball, baseball, soccer, or hockey teams).

35. I think I could have kept my balance while playing rough games (examples: tag, wrestling, karate, judo).

36. I think I could have done activities I usually enjoy for a long time without getting tired out (examples: swimming, jogging, tennis, badminton, rowing, skiing).

37. I think I could have run in a race (example: 100-meter dash).

38. I think I could have worked carefully with my hands (examples: building Lego, making models, sewing, making bead necklaces).

## Supplementary Material

Additional file 1 Translations of the revised CHAQ items into Danish, Turkish, Swedish and Greek language. Click here for file
