# Supplementary material for: Comparing different revisions of the Childhood Health Assessment Questionnaire to reduce the ceiling effect and improve score distribution: Data from a multi-center European cohort study of children with JIA
Source: Pediatr Rheumatol Online J. 2010 May 17;8:16. doi: 10.1186/1546-0096-8-16 (PMC2885385; doi:10.1186/1546-0096-8-16)
Supplement: Additional file 1 — Translations of the revised CHAQ items into Danish, Turkish, Swedish and Greek language. [file 1546-0096-8-16-S1.DOC]

# Translations of additional items to the 30 item CHAQ

### Original items proposed by Lam et al.

31. I think I could have done climbing activities by myself (examples: climbing trees, rocks, or climbing over a fence).

32. I think I could have played team sports with others in my class (examples: basketball, baseball, soccer, hockey).

33. I think I could have played some sports by myself or with a few friends (examples: dribbling and shooting basketball).

34. I think I could have played team sports in competitive leagues (examples: local basketball, baseball, soccer, or hockey teams).

35. I think I could have kept my balance while playing rough games (examples: tag, wrestling, karate, judo).

36. I think I could have done activities I usually enjoy for a long time without getting tired out (examples: swimming, jogging, tennis, badminton, rowing, skiing).

37. I think I could have run in a race (example: 100-meter dash).

38. I think I could have worked carefully with my hands (examples: building Lego, making models, sewing, making bead necklaces).

### Denmark

31. Jeg tror, jeg kan klatre på egen hånd (Eksempler: Klatre i træer, klatre over hegn)

32. Jeg tror, jeg kan være med til holdsport med de andre fra min klasse (Eksempler: Håndbold, fodbold, hockey, basketball – i idrætstimen eller i frikvarteret)

33. Jeg tror, jeg kan være med til nogle sportslege enten alene eller sammen med få andre (Eksempler: Drible med bold i hænder/ ved fødder, spille bold op ad mur, skyde på mål efter basketkurv)

34. Jeg tror, jeg kan deltage i holdsport på konkurrenceplan (Eksempler: Håndbold, fodbold, volleyball, basketball - i en sports-klub)

35. Jeg tror, jeg kan holde balancen under hårde/"vilde" lege (Eksempler: Fangelege, brydning, karate, judo)

36. Jeg tror, jeg kan være med til aktiviteter, jeg normalt er glad for, i længere tid af gangen uden at blive helt udkørt (Eksempler: Svømme, løbe en tur, spille badminton/tennis, cykle)

37. Jeg tror, jeg kan deltage i et kapløb (Eksempel: 100 m sprint)

38. Jeg tror, jeg kan udføre meget præcise ting med mine hænder (Eksempler: bygge med Legoklodser, lave perleplader eller halskæder af perler, sy, bygge figurer i modellervoks / ler)

### Turkey

31. Sanırım, tırmanma aktivitelerini tek başıma yapabilirdim (örneğin ağaçlara, kayalara tırmanma veya çit üzerinden tırmanma).

32. Sanırım, sınıfımdaki diğer kişilerle takım oyunları oynayabilirdim (örneğin basketbol, beyzbol, futbol, hokey).

33. Sanırım, tek başıma veya birkaç arkadaşımla spor yapabilirdim (örneğin top sürme ve potaya basket atma).

34. Sanırım, rekabetin oldugu liglerde takim oyunlari oynayabilirdim (örneğin yerel basketbol, beyzbol, futbol veya hokey takımları).

35. Sanırım, sert sporlar yaparken dengemi koruyabilirdim (örneğin kovalamaca, güreş, karate, judo).

36. Sanırım, genelde keyif aldığım aktiviteleri yorulmadan uzun süre yapabilirdim (örneğin yüzme, koşma, tenis, badminton, kürek çekme, kayak yapma).

37. Sanırım, koşu yarışında koşabilirdim (örneğin 100 metre kısa mesafe koşusu).

38. Sanırım, ellerimle dikkatlice çalışabilirdim (örneğin lego inşa etme, maket yapma, dikiş dikme, boncuklardan kolye yapma).

Sweden

31. Jag tror att jag skulle ha kunnat klättra själv (t.ex. i träd, i bergen eller över staket)

32. Jag tror att jag skulle ha kunnat delta i lagsport tillsammans med mina klasskamrater (ex. basketboll, bandy, hockey)

33. Jag tror att jag skulle ha kunnat delta i någon sport själv eller med några kompisar

34. Jag tror att jag skulle ha kunnat delta och tävla i någon lagsport (t.ex. basketboll, fotboll eller hockey)

35. Jag tror att jag skulle ha kunnat hålla balansen när jag utför tuffa lekar/sporter (t.ex. en knuff i samband med kull, brottning, karate, judo)

36. Jag tror att jag skulle ha kunnat fortsätta med min sport som jag gillar under lång tid utan att bli uttöttad/utmattad (t.ex. simning, joggning, tennis, badminton, skidåkning)

37. Jag tror att jag skulle ha kunnat delta i kapplöpning (t.ex. 60-100 meter lopp)

38. Jag tror att jag skulle ha kunnat utföra pilliga saker med mina händer/fingrar (t.ex. lego, göra modeller, sy, trä halsband)

Greece

31. Νομίζω ότι θα μπορούσα χωρίς βοήθεια να κάνω δραστηριότητες που έχουν σχέση με αναρρίχηση (πχ σκαρφάλωμα σε δέντρα, βράχους ή φράχτες).

32. Νομίζω ότι θα μπορούσα να παίξω σε ομαδικά αθλήματα μαζί με τους συμμαθητές μου (πχ μπάσκετ, ποδόσφαιρο, βόλευ, χάντμπολ)

33. Νομίζω ότι θα μπορούσα να παίζω μερικά αθλήματα μόνος μου ή με λίγους φίλους μου (πχ να κάνω τρίπλες με τη μπάλα ή να σουτάρω στο καλάθι)

34. Νομίζω ότι θα μπορούσα να αγωνιστώ με ομάδες αθλητικών συλλόγων που συμμετέχουν σε πρωταθλήματα (πχ τοπική ομάδα μπάσκετ, ποδοσφαίρου, βόλευ, χάντμπολ)

35. Νομίζω ότι θα μπορούσα να κρατήσω την ισορροπία μου ενώ συμμετέχω σε «σκληρά» παιχνίδια (πχ πάλη, καράτε, τζούντο, ταεκ-βο-ντο)

36. Νομίζω ότι θα μπορούσα να κάνω δραστηριότητες που συνήθως με ευχαριστούν για αρκετή ώρα χωρίς να εξαντληθώ (πχ κολύμπι

37. Νομίζω ότι θα μπορούσα να τρέξω σε αγώνα δρόμου (κούρσα 100 μέτρων)

38. Νομίζω ότι θα μπορούσα να δουλέψω προσεκτικά με τα χέρια μου (πχ να κάνω κατασκευές με «τουβλάκια» τύπου lego, να δημιουργήσω με πλαστελίνη ή πηλό, να ράψω, να κάνω κολιέ λαιμού με χάντρες).

Netherlands

31. Klimmen en klauteren (bijvoorbeeld in een boom of over een hek)

32. Spelen van een teamsport (bv voetbal, basketbal, hockey)

33. Spelen op straat, alleen of met anderen (b.v. dribbelen met de bal, op doel schieten)

34. Spelen van een teamsport in competitieverband (b.v. voetbalwedstrijden, basketbalwedstrijden, hockeywedstrijden)

35. Bewaren van het evenwicht tijdens stoeispelletjes (b.v. tikkertje, stoeien, worstelen, judo, karate)

36. Het voor langere tijd achter elkaar bezig zijn met een leuke activiteit zonder al te moe te worden (b.v. zwemmen, joggen, tennis, badminton)

37. Meedoen aan een hardloopwedstrijdje (b.v. 100 m sprint)

38. Knutselen (b.v. lego bouwen, naaien, kralen rijgen)
